# Comparison of health conditions treated with traditional and biomedical health care in a Quechua community in rural Bolivia

**DOI:** 10.1186/1746-4269-4-1

**Published:** 2008-01-14

**Authors:** Ina Vandebroek, Evert Thomas, Sabino Sanca, Patrick Van Damme, Luc Van Puyvelde, Norbert De Kimpe

**Affiliations:** 1 Institute of Economic Botany, The New York Botanical Garden, Bronx River Parkway at Fordham Road, Bronx, NY 10458, USA; 2 Laboratory of Tropical and Subtropical Agriculture and Ethnobotany, Ghent University, Coupure Links 653, B-9000 Ghent, Belgium; 3 Asociación de Jampiris de Apillapampa, Apillapampa, Provincia de Capinota, Bolivia; 4 Vietnamese Academy of Sciences and Technology, Institute of Chemistry, 18 Hoang Quoc Viet Rd, Cau Giay, Hanoi, Vietnam; 5 Department of Organic Chemistry, Ghent University, Coupure Links 653, B-9000 Ghent, Belgium

## Abstract

**Background:**

The objective of the present study was to reveal patterns in the treatment of health conditions in a Quechua-speaking community in the Bolivian Andes based on plant use data from traditional healers and patient data from a primary health care (PHC) service, and to demonstrate similarities and differences between the type of illnesses treated with traditional and biomedical health care, respectively.

**Methods:**

A secondary analysis of plant use data from semi-structured interviews with eight healers was conducted and diagnostic data was collected from 324 patients in the community PHC service. Health conditions were ranked according to: (A) the percentage of patients in the PHC service diagnosed with these conditions; and (B) the citation frequency of plant use reports to treat these conditions by healers. Healers were also queried about the payment modalities they offer to their patients.

**Results:**

Plant use reports from healers yielded 1166 responses about 181 medicinal plant species, which are used to treat 67 different health conditions, ranging from general symptoms (e.g. fever and body pain), to more specific ailments, such as arthritis, biliary colic and pneumonia. The results show that treatment offered by traditional medicine overlaps with biomedical health care in the case of respiratory infections, wounds and bruises, fever and biliary colic/cholecystitis. Furthermore, traditional health care appears to be complementary to biomedical health care for chronic illnesses, especially arthritis, and for folk illnesses that are particularly relevant within the local cultural context. Payment from patients to healers included flexible, outcome contingent and non-monetary options.

**Conclusion:**

Traditional medicine in the study area is adaptive because it corresponds well with local patterns of morbidity, health care needs in relation to chronic illnesses, cultural perceptions of health conditions and socio-economic aspects of health care. The quantitative analysis of plant use reports and patient data represents a novel approach to compare the contribution of traditional and biomedical health care to treatment of particular health conditions.

## Background

The presence of Western primary health care (PHC) services in rural Bolivia has only gained significance after 1975 when the government increased the number of rural health posts by 80 % over a time period of four years [1]. Therefore, rural communities in Bolivia have always relied heavily on natural resources, especially medicinal plants, for health problems. Worldwide, a similar trend has been observed. According to the World Health Organization, up to 90 % of the population in developing countries relies on traditional medicine (TM) and medicinal plants to meet primary health care needs [2]. In spite of the permanent loss of cultural practices worldwide [3], and also in Bolivia, TM still is very much a part of daily life in the Bolivian Andes [1,4-8]. This is also illustrated by a household survey in an Andean community during which 41 out of 50 participants declared to use TM for treatment of general illness, in spite of the presence of a community PHC service [9].

According to the World Bank, indigenous peoples in Bolivia are among the poorest in poverty analyses, rendering them more vulnerable to disease. As a strategy to improve the health of this group, the Bolivian government has extended the basic health insurance from 1998 (*Seguro Básico de Salud*, abbreviated SBS) to a basic health insurance of indigenous and native peoples (*Seguro Básico de Salud Indígena y Originario*, abbreviated SBSIO) by supreme decree n° 26330 of 2001. The objective of the SBS is to reduce mortality in infants and mothers, as well as the risk, duration and severity of the principal causes of morbidity and mortality in the population. The SBSIO includes among its components the rational use and promotion of the native pharmacopoeia, including medicinal plants. It covers free ambulatory care for conditions that affect children younger than five and pregnant women. Another objective of the SBSIO, although not brought into practice yet, is to reimburse traditional healers for their services and for the natural medicines they provide.

One area of research in medical anthropology has been called the cross-cultural comparative study of human physiological processes, and the way these processes are locally perceived, understood and acted upon. This approach explores the interface between culture and biology, and attempts to compare non-Western or traditional medical systems with biomedicine, inasmuch as this comparison is possible and meaningful [10]. It has been used to examine the physiological dimensions of the Latin American folk illnesses *susto *("fright") and *mal de ojo *("evil eye"), and for assessment of the efficacy of herbal remedies based on combined ethnographic and pharmacological evidence [10-13]. In the present study, we use this approach to make a comparison between health conditions that are treated by a PHC service and traditional healers, respectively, in a Quechua-community in the Bolivian Andes. To this aim, health conditions were ranked according to: (A) the percentage of patients diagnosed with each health condition in the PHC service; and (B) the frequency of plant remedies cited for each health condition from interviews with traditional healers. The underlying assumption is that the citation frequency of plant remedies will parallel the prevalence of health conditions in the study area. This approach is both qualitative (nature of health conditions) and semi-quantitative (ranking of health conditions).

The raw plant use data that is analyzed here is derived from a Spanish field guide co-authored by the traditional healers. This guide consists of 181 plant monographs and contains detailed information about local plant name(s) (in Quechua and Spanish), botanical plant name, medicinal uses reported by healers, preparation and administration of plant species [14]. Healers requested that information related to medicinal plant species and their uses would only be available in a local language publication honoring them as the primary beneficiaries of research. The issue of publication of ethnobotanical information in local languages has recently been discussed by McClatchey and Winter [15]. Although we do not include a table of plant names and uses in this manuscript, primary data are available for verification through the field guide [14]. The analysis we present here makes use of primary plant use data from the guide to calculate the salience of health conditions, hereby making health conditions instead of plants the primary focus of interest. A secondary analysis of plant use data is useful to identify similarities and differences between traditional and biomedical health care, and can indicate what traditional, plant-based medicine has to offer for certain diseases/illnesses [16]. The main research questions of this paper are: "What kind of health conditions are being treated by each health care system?"; and "What is the ranking order of health conditions according to frequency data from patients and plant remedies, respectively?". Although these questions might seem more relevant to medical anthropologists, and less of interest to "classical" ethnobotany studies that describe medicinal plant species and their uses, there clearly exists a need for integrated ethnobotany that embraces questions of relevance to anthropology and addresses public health concerns.

An overview of ethnomedical research in Bolivia consists of studies that focus on the identification of medicinal plants and their uses [6,7,14,17-23] and research into the cultural beliefs and practices related to illness [8,24-26]. Bastien [1,4,27], Alba [28] and Bruun and Elverdam [29] discuss the dual health care system of traditional healers versus biomedicine in Bolivia, and also address the articulation between traditional and biomedical disease classification. However, to the best of our knowledge, no specific data are available from Bolivian communities that cross-link patient data from a PHC service with plant use data from traditional healers.

## Research area

### Climate and vegetation

**Figure 1:**
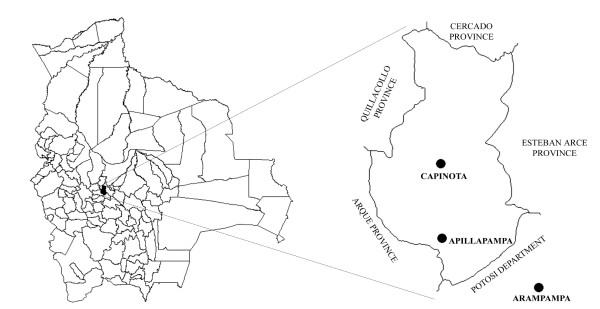
**Map of research area**. Left: the province of Capinota is marked in black within the outline of Bolivia and enlarged at the right. Right: reference points are indicated as black dots and represent the community of Apillapampa, community of Arampampa and the village of Capinota.

**Figure 2:**
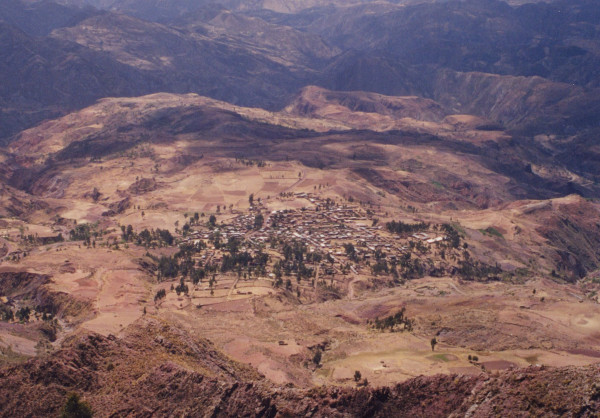
View of Apillapampa from a mountain.

Apillapampa (17° 51'S latitude and 66° 15'E longitude) is a community of around 2500 Quechua-speaking farmers situated in the Bolivian Andes (Capinota Province, Department of Cochabamba) at 3250 meter above sea level and 29 km from the nearest village of Capinota (Figures 1 and 2). Twice a week, a public cargo truck transports people and goods between Capinota and Apillapampa, except during heavy rainfall in the rainy season when the community can become temporarily isolated. Mean annual temperature and precipitation are 18°C and 524 mm, respectively (data from Capinota at 2380 m). In Apillapampa, lower temperatures and a higher precipitation are to be expected due to the higher altitude. During our ethnobotanical study, medicinal plants were collected between 2800 and 3900 m. This range corresponds with two successive ecological units: the *prepuna *(stretching out between 2000–2300 and 3100–3300 m) and the *puna *(between 3100–3300 and 3900–4000 m) [30]. The vegetation of the research area has undergone significant human influence and is therefore mainly secondary in nature and characterized by shrubs of different height (called *chaparrales *and *matorrales*) and grasses [31]. In the upper part of the *prepuna*, between 2600–2700 and 3100–3200 m, climax vegetation free of anthropogenic disturbances is formed by *Kageneckia lanceolata *Ruiz & Pav. and *Schinus molle *L.. In soils with a high degree of erosion this vegetation is substituted by *Dodonaea viscosa *Jacq. and *Baccharis dracunculifolia *DC. (*matorrales *of 1–2 m). Associated species include *Salvia haenkei *Benth., *Lycianthes lycioides *(L.) Hassl., *Aloysia gratissima *(Gillies & Hook.) Tronc., *Cheilanthes myriophylla *Desv., *Kentrothamnus weddellianus *(Miers) M.C. Johnst. and *Buddleja tucumanensis *Griseb.. In the *puna *part of the study area, between 3200 and 3900 m, the climax vegetation consists of *Polylepis besseri *Hieron. and *Berberis commutata *Eichler. This vegetation is substituted by grasslands containing *Astragalus *sp.*, Peperomia peruviana *(Miq.) Dahlst., *Hypseocharis pimpinellifolia *J. Rémy, *Satureja boliviana *(Benth.) Briq. and *Relbunium *aff.*ciliatum*. A more detailed listing of plant species of the *prepuna *and *puna *in Bolivia is given in [30]. An account of plant species communities in the adjacent Capinota area, physically close to Apillapampa but situated lower in altitude, is given in [31]. The most common plant species of the lower Andean valleys with an altitude up to 3200 m are described in [32]. Pestalozzi [7] presents a flora of *Majasaya mujlli*, a *puna *community in the La Paz department that is situated higher than Apillapampa (between 3800 and 4500 m). The flora of Apillapampa has particular species in common with each of these reference works.

### Subsistence

**Figure 3:**
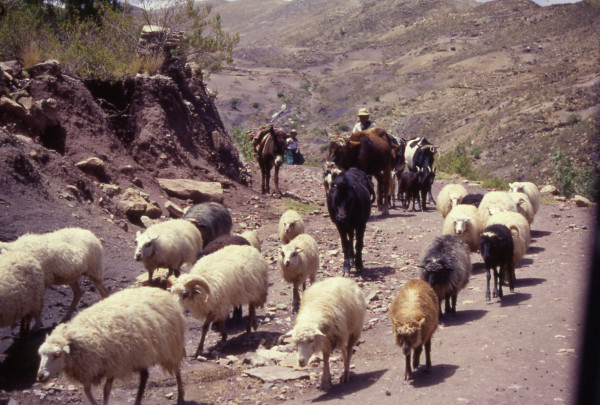
Herding of sheep, goats and cows in Apillapampa.

Farmers in Apillapampa herd sheep, goats and cows and still practice subsistence agriculture on sloping land with fields at different altitude levels in order to diversify production (Figure 3). Potatoes, *oca *(*Oxalis tuberosa *Molina; Oxalidaceae), wheat, corn and barley are grown for local consumption, barter and seeds. Strong erosion, resulting in degraded soils and scarce availability of cropland, is responsible for limited crop production [33]. As a result of the weak economic situation, the community suffers from constant emigration. Since essentially men are migrating, labor pressure on women who remain behind is high. Consequently, some traditional tillage practices are abandoned, which further results in soil degradation and yield decreases. This in turn contributes to poor diet and increased vulnerability to disease.

### Health care

**Figure 4:**
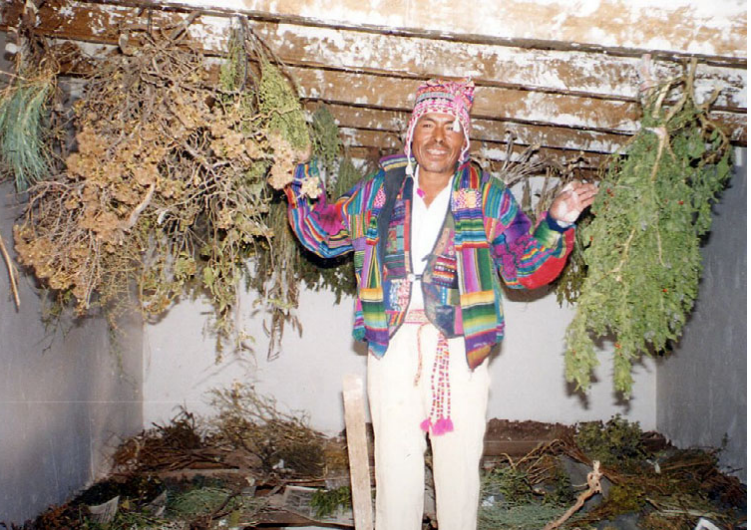
**Don Sabino Sanca, President of the traditional healers' organization***Asociación de Jampiris de Apillapampa *showing dried medicinal plants.

Limited biomedical health care in Apillapampa is provided since 1985 by a local NGO (Fepade), and consists of vaccinating children, provision of mineral supplements to pregnant women, and ambulatory care with antibiotics, painkillers, rehydration salts, and spasmolytic, anti-inflammatory and anthelminthic drugs. A Spanish and Quechua speaking medical doctor and assistant nurse originating from outside the community staff the PHC service on weekdays. During weekends, a female traditional healer from the community is attending, who is also trained in basic practices of Western medicine. Consultation is free, but adults and children older than 5 have to pay for medicines. For surgery, patients are referred to the nearby village of Capinota, or to the city of Cochabamba. TM in Apillapampa is offered by traditional healers. Eight healers (seven men and one woman) are united in a semi-formal organization, called *Asociación de Jampiris de Apillapampa *(formerly named *Asociación de Médicos Tradicionales*) that was founded in 1994. The healers are active in their own community (Figure 4), as well as in more urban zones near the city of Cochabamba. Knowledge and hands-on experience are exchanged among them on a regular basis during workshops or *talleres*.

## Methods

### Methodological considerations

The methodology used in the present study obviously does not allow for a direct quantitative comparison of data since we did not have patient data from healers. Healers did not use a patient logbook, and there exist cultural barriers to share hands-on treatment of patients with outsiders. Therefore, plant use data were taken as an indirect measure of the local prevalence of health conditions by assuming that plant remedies are experimentally selected by local people as a dynamic response to threats on the human physiological system.

### Agreements and permits

Before the start of research, a written agreement between the traditional healers and researchers, and a fieldwork permit from the Bolivian Ministry of Sustainable Development and Planning in La Paz (*Directorio General de Biodiversidad*) were obtained. The *Centro de Biodiversidad y Genética *from the *Universidad Mayor de San Simón*, Cochabamba, was the local partner supporting permit application.

### Ethnobotanical Fieldwork

Ethnobotanical fieldwork took place in the dry (July to October) and rainy (January to April) season of 2000–2001. During a total of thirty field trips in the company of a traditional healer three to four voucher specimens of each plant species were collected according to botanical standards. Vouchers were identified by the authors and local specialists and deposited in the *Herbario Nacional Forestal Martin Cárdenas *in Cochabamba (BOLV) and the *Herbario Nacional de Bolivia *in La Paz (LPB). Semi-structured interviews were conducted in the community with individual healers and voucher specimens were used as a prop for interviewing. Personal background characteristics of the healers, seven men and one woman, are given in [34]. Interviews took place in Spanish. Healers were asked details about vernacular plant names, medicinal uses, disease etiologies, plant parts used, preparation, mode of administration, dosage, treatment regime, and possible use-restrictions (for pregnant women and children).

In 2003, separate structured interviews consisting of multiple choice questions were held individually with five healers (four men and one woman) about the cost of treatment with traditional medicine. Each healer was asked the following questions: (A) What kind of illnesses do you treat (only spiritual illnesses, only physical illnesses, or both spiritual and physical illnesses)?; (B) Do patients have to pay for treatment (yes or no)?; (C) Do they have to pay for medicinal plants (yes or no)?; (D) How do patients pay (labor exchange, animals, crops, or money)?; (E) How much is payment (less than 5, between 5 and 20, between 20 and 50, more than 50 Bolivian Boliviano, or depending upon the type or duration of treatment)?; (F) When is payment due (immediately after consultation, or later when the patient has regained health)?

### Analysis of patient data from the community PHC service

The logbook of the community PHC service was consulted for morbidity data that covered the two periods of fieldwork (July to October 2000 and January to April 2001). Data from 324 patients were entered into an Excel spreadsheet, with rows representing individual patients and columns representing data on age, sex, and disease diagnosis in Spanish. Analysis consisted of calculating the age-distribution of patients, and the frequency of each ailment based on the number of patients.

### Analysis of plant use data from healers

Interview data were organized in an Excel spreadsheet with each row representing a plant use report for a particular species (e.g. use of *Chenopodium ambrosioides *L. for treating colic [14]) and columns representing data on voucher number, local Spanish and/or Quechua plant name, scientific plant name, plant family, Quechua name of illness, Spanish name of illness, and the total number of responses from the eight healers for that particular use-report. Each individual plant use-report confirmed by another healer increased the number of responses. For instance, all healers use *C. ambrosioides *to treat colic, which brings the number of responses up to eight [14]. Next, data related to individual health conditions was extracted from this spreadsheet to calculate proportions based upon the cumulative number of responses of all plant use data for each health condition.

### Comparison of health conditions between the community PHC service and healers

Upon comparing health conditions from the PHC service and healers, we used as much as possible the original denomination of these ailments. For instance, percentages related to scabies were calculated directly for both health care systems, without first lumping them together with other reports of insect stings and infestations. However, in some cases it was not possible to avoid grouping health conditions. For instance, the PHC logbook contained patient records of both "pneumonia" and "respiratory infection". Likewise, healers reported plant remedies for "pneumonia" and "lung problems". Therefore, we created a new category of "respiratory infections" that grouped these unspecified respiratory problems together with pneumonia, cough, otitis and sore throat. A few other health conditions were lumped for the same reason (see Table 1).

**Table 1:** Local names and description of health conditions listed in figure 5 according to traditional healers

Health condition	Spanish or Quechua name (the latter name is underlined)	Diagnostic symptom(s) of health condition according to healers
arthritis	reumatismo, t'ullu taxaj	Pain in bones and knees during low environment temperature
biliary colic and cholecystitis	cólico, cólera, colerina, bilis, jayaq'e punkiy, jayaq'in	Reference to the gall bladder, (cramping) abdominal pain, yellow or green-colored vomiting, sharp taste in the mouth, flatulence. Caused amongst others by feelings of anger towards family. Headache, acidity and heartburn can be accompanying symptoms
cold, flu and chills	resfrío, gripe, ilasqa, chiri pasasqas	Elevated body temperature, sneezing, phlegm. Chiri pasasqas: chills caused by working at night in a cold and/or wet environment
conjunctivitis	ch'ojñi, ñawi	Eye feels warm and painful, inflammation, eye cannot be opened, pus, blurred vision
constipation	estreñimiento, empacho	Inability to release stool, need to apply enema
contraception	planificación familiar, warmis	Women who do not want to have more children
diabetes	diabetes	Patient has dull abdominal pain, sweet tasting urine, a dry mouth and a desire to eat sweets
diarrhea	diarrea	Linked to intestines, can be associated with vomiting and stomachache
fever	k'aja, junp'iy	Dry mouth and nose, elevated body temperature (patient feels 'hot'), accelerated pulse, headache
fractures and sprains	fracturas, torceduras	A distinction is made between closed and open fractures
intestinal parasites	gusanos en los intestinos, botar bichos de la barriga, limpiar las tripas	Worms that have to be expelled from the intestines
labor and puerperium	parto	Labor induction, pain, bleeding, recovery from childbirth
*madre *("rupture")	madre, quebradura, quebración, chullchusqas	Caused by working hard or lifting heavy objects (and not eating well). Abdominal pain, flatulence, desire to constantly go to the bathroom, blood in stool (blood in urine is mentioned as well), vomiting and dizziness, diarrhea is possible, itchy hands and feet, difficult urination, loss of appetite, unwillingness to work, weight loss, "lack of blood", loss of vitality
*maldición *("cursed")	jap'eqa (asustarse, manchariy); maldición, hechizo, brujería	Caused by fright or witchcraft; the patient does not cure, shivers and has pain in the entire body, abdominal pain, headache, desire to sleep, unwillingness to work, loss of appetite, weak pulse, sadness, fever is possible. In the case of *maldición *the patient already knows from his dreams that he is cursed
pain, excluding toothache	nanay	Including headache, abdominal, body, back and chest pain
respiratory infections, including cough, otitis, pneumonia and sore throat	cough: tos, ch'ujchu; otitis: sordo, dolor de oído, ninri jukara; pneumonia: neumonia, costa(d)o	Cough can be "dry" or "wet" (with phlegm) and accompanied by a sore throat and fever; otitis is reported as deafness/inability to hear, and earache with yellow pus; pneumonia symptoms include cough, (high) fever, and chest pain and its equivalent costa(d)o consists of cough with blood, and pulsating chest pain
scabies	sarna, rasca paloma, rasca rasca	Itching and infecting skin eruptions caused by scabies
stomachache and gastritis	dolor de estomago, gastritis, bultos en el pecho, dolor de estómago y corazón	Heartburn (described as sensation of "stones" or burning feeling in chest), vomiting, pain in higher abdomen, loss of appetite, stomach problems are thought to be caused by eating burned food or drinking too much alcohol
toothache	dolor de muelas, dolor dedientes	Painful teeth
urijasqa ("malnutrition")	urijasqa	Illness caused in children by the bad smell of a dead animal; symptoms include swollen and painful belly, weight loss, growth retardation, yellow colored skin
urinary and kidney problems	quebración, jisp'ay p'iti quebradura, hemorragia, mal de riñon, dolor de riñones	These can include difficult or painful urination; reduced urination, a "desire to go to the bathroom all the time", painful abdomen, dark colored urine; blood in urine; "cold" bladder, back pain, and swollen hands and feet
*wayra *("evil wind")	aire, wayra, wayra jap'isqa	Symptoms can range from stiff and painful muscles to (facial or even corporeal) paralysis, depending on the degree of severity according to healers. In case of facial paralysis, the mouth is twisted or an eye-lid is drooping
wounds and bruises	herida, hinchazón, moradura, punkisqa, abceso, ch'upu	Include abscesses (a swelling with pus that does not want to open), cuts, bleeding from the skin. Bruises are described as a localized feeling of heat, swelling of the skin without pus

## Results and Discussion

### Patient data from the community PHC service

**Figure 5:**
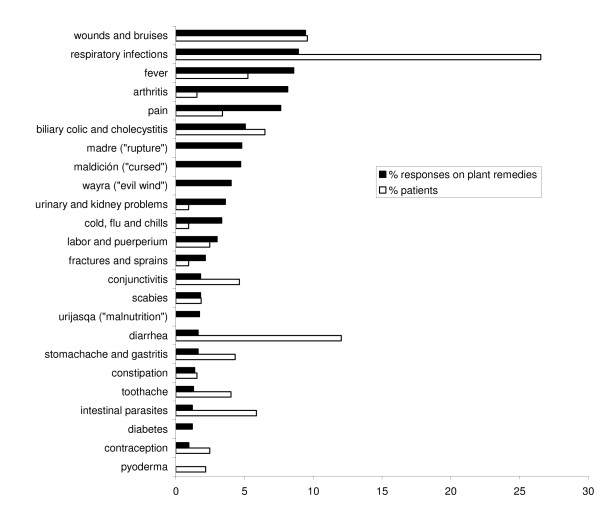
**A comparative ranking of health conditions in the biomedical and traditional health care system in Apillapampa based on patient data from the primary health care service (n = 324 patients) and the citation frequency of plant use reports from eight traditional healers (n = 1166 responses)**. For healers' diagnostic criteria of these illnesses (except pyoderma) see table 1.

Figure 5 combines two graphs that represent the salience of ailments according to: (A) patient data from the PHC service (% patients); and (B) the percentage of plant use reports from traditional healers. The graph that represents data from the PHC service covers 94% of all patients who had a consultation during the selected time periods. Health conditions diagnosed in less than five patients were excluded if no comparative data on those conditions was available from traditional healers. The most frequently reported diseases or symptoms in the PHC service (diagnosed in at least 5% of patients) are: (A) respiratory infections; (B) diarrhea; (C) wounds and bruises; (D) biliary colic and cholecystitis; (E) intestinal parasites; (F) fever; and (G) conjunctivitis (Figure 5).

**Figure 6:**
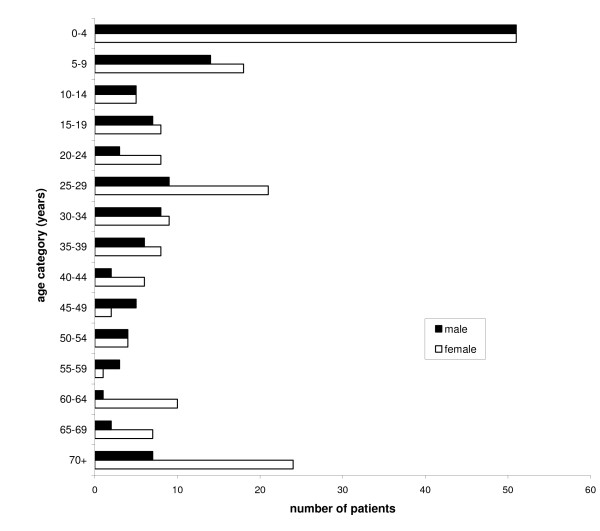
**Number of male and female patients grouped according to age category in the sample from the primary health care service in Apillapampa**. Combined data from July-October 2000 (dry season) and January-April 2001 (rainy season) (N = 324 patients).

Depending on the type of ailment, the number of patients in the community PHC service varied between 1 and 45. Figure 6 shows the number of male and female patients according to age category (n = 191 and 129, respectively). The largest age group are children younger than five (n = 103; one third of patients). Other important categories (around 10 % of patients each) are children between 5–9, adults between 25–29, and people older than 70. The high number of women in age group 25–29 can be partly attributed to labor and puerperium. Children younger than five mainly suffer from diarrhea, usually in combination with intestinal parasites, or from a respiratory infection, including pneumonia (40 and 30 % of cases, respectively). Forty eight percent of all respiratory infections in the PHC service are diagnosed in children younger than five, and this age group also covers 41 % of all gastrointestinal disorders. All cases of cholecystitis (n = 9) are diagnosed in people older than 65.

### Plant use reports from traditional healers

Interviews with healers about 181 medicinal plant species generated a total of 1166 responses. The cumulative number of responses for individual health conditions varied from 1 (e.g. gangrene, cholera) to 100 (fever). The range of the number of plant species cited per ailment was 1 to 61. Ailments with less than 1% of all responses were omitted from analysis if insufficient corresponding patient data related to these ailments was available from the community PHC service. In Figure 5, the graph that represents the citation frequency of plant remedies covers 88% of all responses.

According to Figure 5, plant remedies mentioned most frequently by healers (each constituting at least 5% of all responses) are used to treat: (A) wounds and bruises; (B) respiratory infections; (C) fever; (D) arthritis; (E) pain (body pain, abdominal pain, headache and/or back pain); (F) biliary colic; (G) *maldición *("cursed"); and (H) *madre *("rupture"). The latter two conditions may be called folk illnesses based on their possible association with several biomedical diseases. *Maldición *is a persistent illness with a variety of symptoms that refer to a general state of malaise. *Madre *or *quebradura *("rupture") is an illness that healers associate with heavy labor on agricultural fields. While several of its symptoms correspond with the biomedical definition of a hernia, it is probable that this illness is associated with megacolon and intestinal volvulus (abnormal twisting of the intestine) occurring in Chagas' disease or American trypanosomiasis, a serious human parasitic disease [28,35,36]. Alba [28] further illustrates that multiple biomedical etiologies may underlie *quebradura *by linking it to a prolapsed uterus or vagina in his Bolivian study area.

Table 1 presents healers' diagnostic symptoms of the health conditions listed in Figure 5. The table shows that healers are to some degree familiar with Western concepts of disease. For instance, healers' diagnostic symptoms of biliary colic, gastritis and diabetes match the scientific description of symptoms. In addition, healers themselves indicated that *urijasqa *is related to malnutrition. *Urijasqa *is an illness believed to originate from inhalation of bad odors that are transmitted from a dead animal to small children which results in a swollen belly and developmental retardation. Table 1 also provides insight into local beliefs about illness causation. Factors mentioned as causes of illness include physical (food, alcohol, environmental temperature and humidity, labor), social (feelings of anger), as well as spiritual (witchcraft, bad odors, evil wind) factors.

### Comparison of health conditions treated by biomedical and traditional health care in Apillapampa

Data from the PHC service and healers related to 50 and 67 different health conditions, respectively. Sixty-six percent of health conditions from the PHC service overlap with those reported by healers, while the overlap is 49% for the list of health conditions from healers. Figure 5 shows that respiratory infections, wounds and bruises, fever and biliary colic/cholecystitis rank high on both graphs from the PHC service and healers. Hence these health conditions are substantially covered by both health care systems. On the other hand, only few patients (6 on a total of 324) were treated in the PHC service for arthritis, while healers frequently report plant remedies for rheumatism (the total number of responses and the number of different plant species used for arthritis is 95 and 56, respectively). Although low in citation rate, healers also mentioned plant remedies for other chronic conditions, such as diabetes, skin allergy, prostate problems, liver cirrhosis and depression, while there were no reports of these health conditions in the PHC service logbook for the selected time periods. Hence, traditional health care appears to be complementary to biomedical health care for chronic illnesses. Another domain where TM offers predominant care is for folk illnesses such as *maldición*, *madre*, *wayra *and *urijasqa *that are particularly relevant within the local cultural context.

Our results also demonstrate the importance of the community PHC service in providing health care to children younger than five who frequently suffer from diarrhea, intestinal parasites and respiratory infections. These conditions have been identified as the main causes of child morbidity and mortality in Bolivia [37]. Healers' knowledge of plant remedies to treat diarrhea and intestinal parasites is not as diversified as their knowledge about plants for treating other health conditions such as respiratory infections. The total number of responses was 19, 14 and 104 for diarrhea, intestinal parasites and respiratory infections, respectively. Hence, there are relatively few reports of plant remedies to treat the former two health conditions. It is possible that patients prefer to use the PHC service for diarrhea and intestinal parasites. Since the basic health insurance guarantees free treatment for children younger than five in the PHC service, mothers may prefer biomedical over traditional health care for their young children. However, the exact contribution of TM in treating these conditions can only be clarified by future studies that compare patient data between the PHC service and healers.

In a previous study in Apillapampa, an equal number of participants reported using medicinal plants and pharmaceuticals for treating health conditions [9]. The study asked two general separate questions "when you are ill, do you use medicinal plants" and "when you are ill, do you use pharmaceutical products like antibiotics, aspirin, ..." and hence did not query about the preferred, sequential or simultaneous use of medicinal plants and pharmaceuticals. Participants most often obtained pharmaceuticals from the PHC service as compared to the nearest village of Capinota or the main city of Cochabamba. Out of 50 interviewed community members, four declared to use medicinal plants preventively to improve their general health status [9]. Although the present study did not focus on the preventive use of medicinal plants, occasionally healers would mention about a particular plant that it "contains vitamins", "cleanses or purifies the body", or "gets rid of filth/dirt". We did not observe that the PHC service and traditional healers exchanged information about shared patients, but the medical doctor in charge of the PHC service had a very positive attitude towards TM and even treated some of his patients with products prepared by the traditional healers (including a cough syrup, an ointment for muscular pain, and eye drops for conjunctivitis). Also, healers were unanimously positive about biomedicine [34]. However, it should be added that the present study took place before an incident in April 2004 during which three children died from a vaccine that was prepared and administered by biomedical staff from the hospital of the neighboring village of Capinota using a muscle relaxant instead of physiological solution. According to local news coverage, after the event community members rejected the presence of biomedical "outsiders" and a new PHC service in their community [38].

Pyoderma is a specialized biomedical term used to denominate any purulent skin disease. It is reported in the PHC service in addition to scabies, a skin condition caused by the microscopic mite *Sarcoptes scabiei*. Healers also report scabies, as well as other skin conditions, including skin allergy (9 responses), fungal skin infections (8 responses), flea infestations (3 responses), and eruptions caused by the bug *jallp'a *(3 responses). Hence, healers tend to over-differentiate in their classification of skin conditions as compared to the biomedical health care system, meaning that they report several different ailments that may correspond with pyoderma. However, the exact biomedical correlates of each of these skin conditions remain unknown and therefore they were not paired with pyoderma in Figure 5.

### Comparison with morbidity data from literature

The prevalence of health conditions in Apillapampa according to the graphs in Figure 5 corresponds well with local and regional morbidity data from literature. A diagnostic guide from the community NGO shows that babies and adolescents in Apillapampa frequently suffer from diarrhea, respiratory infections, skin infections (pyoderma and scabies), and eruptive diseases (e.g. chicken-pox). In the adult population, respiratory, parasitic, hepato-biliary (colic), and degenerative diseases like arthritis are prevalent, as well as Chagas' disease and tuberculosis [33]. Chagas' disease remains a serious public health problem in Bolivia and the dry Interandean Valleys of the Cochabamba Department to which our study area belongs is considered the center of dispersion of the Chagas' parasite vector [39]. Chagas' disease consists of an acute and a chronic phase. The mean age of acute and chronic infection in endemic areas is 4 and 35–45 years old, respectively [40]. Both phases can be free of symptoms or life threatening. The acute phase usually occurs unnoticed because it is symptom free or exhibits only mild symptoms which are not unique to Chagas' disease. Symptoms can include prolonged fever, fatigue, body aches, anemia, headache, rash, loss of appetite, diarrhea, and vomiting. Most patients experience mild clinical symptoms. Only a minority develops severe clinical syndromes, including meningoencephalitis, heart dilation and heart failure, and about 10 % dies from these disorders. A number of patients may develop chronic symptomatic systemic Chagas' disease decades after the acute stage, even when no detectable traces of the parasites are any longer encountered in the blood. The clinical picture of Chagas' disease is dominated by cardiac and digestive disorders and destruction of enteric nerves [39,40]. It is remarkable that our research data did not provide any direct mention of Chagas' disease in spite of its prevalence in the community. The first author was able to directly observe a well-recognized marker of Chagas' disease that appears in some patients during the acute phase, the unilateral periocular swelling (called Romana's sign). In order to diminish the occurrence of infection, the local NGO Fepade has been actively campaigning in the community to educate community members about ways to eradicate nesting sites of the vector, among others by modifying people's living environment from adobe houses with thatch roofs to painted walls and artificial roofing. However, in spite of clear health dangers imposed by this disease, no direct patient data related to Chagas' disease where recorded from the PHC service, and only one healer reported a plant remedy for the disease. Several reasons can explain this. No routine screening facilities for blood, cardiac or gastrointestinal testing are available for this disease in the community PHC service. Moreover, clinical symptoms may differ in severity, and cardiac and digestive disorders may well be caused by other pathologies. Therefore, patients diagnosed with symptoms such as constipation and intestinal obstruction may include those who suffer from the disease but who remain unnoticed due to absence of diagnostic testing. Likewise, healers reported remedies for constipation. Also, several symptoms listed in Table 1 for "*madre*" (rupture) clearly refer to a chronic gastrointestinal condition related to intestinal obstruction, which may point to a correspondence with Chagas' disease. Healers scarcely mentioned plant remedies to treat a "heart problem" but did not make any spontaneous reference to Chagas' disease at the time being. It may be that healers are not familiar with the terminology and biomedical concept of Chagas' disease. In addition, assuming that "*madre*" corresponds with Chagas' disease, healers attribute a different causal factor to the disease (performing hard labor on the land as opposed to transmission of the parasite by an insect vector).

Bastien [4] lists colic as an important illness of the gastrointestinal tract in the Bolivian Andes. This author also mentions that rheumatoid arthritis tends to be common among adults in the Bolivian Andes, especially in older women who work on the land in skirts while cold wind quickly cools off their legs. Furthermore, Andeans frequently suffer from conjunctivitis due to traveling in open trucks over dirt roads and cooking in huts without chimneys [4]. In an Aymara high altitude community in Bolivia the following folk illnesses and health conditions were reported as important: (a) *incuria *or *quebración *('rupture', with etiology and symptoms in common with *madre *in this study); (b) cough; (c) sunstroke; (d) *ch'exori *or *makhurja *(according to local diagnosis symptoms are exhaustion, fever and headache caused by working hard and enduring cold temperatures); (e) gall bladder problems (probably related to colic); and (f) diarrhea [7]. In another study in Bolivia the (folk) illness most frequently reported by Andean community members was *pisti unquy*, which corresponds with flu or common cold according to a local health care worker. Abdominal pain ranked second and *wayra *ranked fifth. The author also presents morbidity data for the region, which is larger and lower in altitude (1700 to 3450 m) than our own study area. Trauma was the most prevalent pathology, followed by sexually transmitted diseases, gastrointestinal illnesses, malaria, skin conditions and respiratory infections [28]. All these studies are local language publications.

A review of the English scientific literature yielded no morbidity data for specific Bolivian communities. A PAHO (Pan-American Health Organization) report lists the ten principal causes of general morbidity in Bolivia in 1993 [41]. However, this report does not provide morbidity data according to biogeographic region (e.g. Andes versus Amazon), which is important because each region exerts its own stresses upon human physiology. Alba [28] demonstrates that farmers from high altitude areas have great difficulties to maintain their health equilibrium, as was evident from the fact that the majority (94%) of interviewed families reported at least one sick family member over the past year. Andeans are confronted with hypoxia and hypothermia, stresses that are related to the altitude, and malnutrition [1,41]. Morbidity data from Andean communities point to a high degree of respiratory disorders which are the most common complaint at dry, higher altitudes [42]. Gastrointestinal (diarrhea, gastritis) and musculoskeletal disorders (rheumatism) rank second or third [43,44]. In southern Peru, this ranking also corresponded well with the local citation frequency of plant remedies [43]. Although digestive disorders tend to be more frequent at lower altitudes, they are also part of the main serious diseases in adults from highland communities. Musculoskeletal disorders appear to be associated with an agricultural mode of production [44].

### Folk illnesses and their biomedical correlates

It is well-known from literature that folk-illnesses usually do not have a direct one-to-one correspondence with discrete diseases within the biomedical system. This is also evident from Table 1 that lists healers' diagnostic symptoms of folk illnesses such as *madre*, *maldición *and *wayra*. *Wayras *are wind- or airborne diseases that can cause symptoms varying from stiff muscles to (facial) paralysis, including Bell's Palsy [4]. Alba [28] confirms that *wayra *is equivalent to neuralgia, neuritis and paralysis. Our results further suggest that folk illnesses are not just mere psychological illnesses, as has been often assumed in the literature [10], but may represent *real *medical problems with a variety of physiological symptoms, including, fever, headache, abdominal pain, lack of appetite, nerve paralysis, and muscle cramps. In a study on two Mexican folk illnesses called *susto *and *mal de ojo*, it was found that the majority of symptoms were worthy of medical attention and possibly life threatening if not treated [11]. Further follow-up research into the relationships between folk illnesses and biomedical diagnoses is necessary to improve culturally-sensitive health care by promoting the dialogue between patient and health care provider. One approach would be to conduct interconsultations of patients by a traditional healer and a biomedical health care provider in order to cross-link their diagnoses as described by Alba [28]. The importance of the folk illness *mal de susto *is also recognized in the SBSIO from the Bolivian government that stipulates that this illness merits primary health care.

### Cost of treatment: biomedical versus traditional medicine

In the PHC service, patients do not have to pay for consultation but they do have to pay for prescribed medicines, with the exception of children younger than 5 who receive free medication thanks to the SBS. Payment is charged per tablet. Prices of some prescriptions are as follows (data from April 2003): cotrimoxazol for adults and metronidazol: 0,50 BOB (0,0625 €) per tablet; cotrimoxazol for infants and aspirins: 0,20 BOB (0,025 €) per tablet. Rehydration salts are provided free of charge. Payment on credit is allowed. Sometimes the medical doctor performs follow-up visitations of patients at home.

Table 2 presents results from interviews with five healers about the cost of treatment. Healers treat diseases of physical and spiritual origin, with the exception of the only female healer we interviewed who exclusively deals with naturalistic diseases. Patients usually have to pay for treatment. Sometimes they also have to pay for medicinal plants, although a female healer stated that poor patients do not have to pay at all and can collect the required medicinal plants themselves. Another healer stated that payment depends on the patient. In Apillapampa, payment is usually in cash, but other means, e.g. by labor exchange, animal(s) (products), and crops are also possible. Exchange of goods or labor is in agreement with traditional Andean principles of reciprocity [1]. Total amounts to be paid to healers for curing in Apillapampa ranged between 2–20 BOB (1 BOB = 0,125 €). In the city of Cochabamba, healers charge between 5–100 BOB (data from April, 2003). Payment can be requested after consultation, or after the patient has regained health.

**Table 2:** Cost of traditional health care and payment modalities according to five traditional healers

	**Healer**				
	1	2	3	4	5
**Type of illnesses treated:**					
spiritual					
physical	X				
both spiritual and physical		X	X	X	X

**Patients have to pay for:**					
treatment	d	d	X	X	X
medicinal plants	d	d	X	X	

**Means of payment:**					
*ayni *(labor exchange)		X			
animals		X			
crops		X	X		
money	X	X		X	X

**Amount to be paid (in BOB):**					
< 5					
5–20		X	X	X	X
20–50					
> 50					
depends on treatment (type, duration)	X		X		

**Payment is due:**					
immediately after consultation	X	X	X		X
later, when patient has regained health	X	X		X	X

Explanation of symbols: X: answer indicated by healer; d: healer stated that it depends on the patient's financial situation; BOB: Bolivian Boliviano; 1 BOB = 0,125 € in April 2003

The fact that plant remedies are directly available from the surroundings might be a good strategy to avoid costly long-lasting biomedical care, especially for chronic health conditions. Although consultation with traditional healers is not necessarily free of charge, healers handle more flexible payment modalities as compared to biomedical health care where tablets have to be purchased with cash. Healers may exempt poor patients from payment. Alternatively, they may agree that the patient has to pay after the desired effect of treatment has been demonstrated. Outcome-dependent compensation of healers has been observed elsewhere and appears to be one of the reasons why patients prefer to consult healers even with the expansion of biomedical health care [45].

## Conclusion

The present study looked for similarities and differences between traditional and biomedical health care. Treatment offered by TM in the Andean study area both overlaps with and complements biomedical health care. Not only do healers know plant remedies for the majority of health problems treated by the community PHC service, but they are also major health care actors for treating arthritis, a major chronic disorder that prevails in the community, and several folk-illnesses. These folk-illnesses probably correspond with several other persistent, long-lasting or chronic health conditions, including malnutrition (*urijasqa*), Bell's Palsy (*wayra*) and Chagas' disease (*madre*). The exact biomedical nature of these conditions remains to be studied. The unique position of TM for exclusive treatment of folk-illnesses remains largely unchallenged in present day rural Bolivia. On the other hand, our study also demonstrated the merits of the PHC service in the community in providing health care to children under five for health conditions that have been recognized as the main causes of child morbidity and mortality worldwide (diarrhea and respiratory infections). Since children under five are the age group most vulnerable to disease, the direct contribution of TM to child health care in Apillapampa should be elucidated in future studies that provide a direct comparison between patient data from the traditional and biomedical health care system..

From our results, we can infer that TM is both "traditional" and "modern". TM is traditional in the sense that it is in agreement with and inclusive of local belief systems and explanatory models of illness. At the same time, TM is "modern" or "adaptive" for being all-round and dealing with a diverse array of local health conditions. TM is also "practical" in its flexible payment modalities. We believe that these three unique features (inclusiveness, all-roundness and flexibility) contribute to TM's continued popularity in the community, even in the presence of a community PHC service.

## Competing interests

The author(s) declare that they have no competing interests.

## Authors' contributions

IV designed the research study, conducted fieldwork and drafted the manuscript. ET and SS assisted with fieldwork. PVD, LVD and NDK supervised research activities and provided comments on the draft of the manuscript. All authors read and approved the final manuscript.
